# Public Perceptions of Very Low Nicotine Content on Twitter: Observational Study

**DOI:** 10.2196/63035

**Published:** 2024-12-04

**Authors:** Zidian Xie, Xinyi Liu, Xubin Lou, Dongmei Li

**Affiliations:** 1 Department of Clinical and Translational Research University of Rochester Medical Center Rochester, NY United States; 2 Goergen Institute for Data Science University of Rochester Rochester, NY United States

**Keywords:** very low nicotine, Twitter, public perception, observational study, content analysis

## Abstract

**Background:**

Nicotine is a highly addictive agent in tobacco products. On June 21, 2022, the US Food and Drug Administration (FDA) announced a plan to propose a rule to establish a maximum nicotine level in cigarettes and other combusted tobacco products.

**Objective:**

This study aimed to understand public perception and discussion of very low nicotine content (VLNC) on Twitter (rebranded as X in July 2023).

**Methods:**

From December 12, 2021, to January 1, 2023, we collected Twitter data using relevant keywords such as “vln,” “low nicotine,” and “reduced nicotine.” After a series of preprocessing steps (such as removing duplicates, retweets, and commercial tweets), we identified 3270 unique noncommercial tweets related to VLNC. We used an inductive method to assess the public perception and discussion of VLNC on Twitter. To establish a codebook, we randomly selected 300 tweets for hand-coding, including the attitudes (positive, neutral, and negative) toward VLNC (including its proposed rule) and major topics (13 topics). The Cohen κ statistic between the 2 human coders reached over 70%, indicating a substantial interrater agreement. The rest of the tweets were single-coded according to the codebook.

**Results:**

We observed a significant peak in the discussion of VLNC on Twitter within 4 days of the FDA’s announcement of the proposed rule on June 21, 2022. The proportion of tweets with a negative attitude toward VLNC was significantly lower than those with a positive attitude, 24.5% (801/3270) versus 37.09% (1213/3270) with *P*<.001 from the 2-proportion *z* test. Among tweets with a positive attitude, the topic “Reduce cigarette consumption or help smoking cessation” was dominant (1097/1213, 90.44%). Among tweets with a negative attitude, the topic “VLNC leads to more smoking” was the most popular topic (227/801, 28.34%), followed by “Similar toxicity of VLNC as a regular cigarette” (223/801, 27.84%), and “VLNC is not a good method for quitting smoking” (211/801, 26.34%).

**Conclusions:**

There is a more positive attitude toward VLNC than a negative attitude on Twitter, resulting from different opinions about VLNC. Discussions around VLNC mainly focused on whether VLNC could help people quit smoking.

## Introduction

Over a billion people worldwide smoke cigarettes [1]. In 2021, around 46 million (18.7%) US adults reported the use of any tobacco product, including cigarettes (11.5%), e-cigarettes (4.5%), etc [2]. Cigarette smoking is known to lead to many health symptoms or diseases, such as asthma, chronic obstructive pulmonary disease (COPD), and lung cancer [3,4]. As the leading cause of preventable death and disease in the United States, it was estimated that approximately 480,000 deaths annually are related to cigarette smoking [5]. In addition, tobacco use has a substantial financial burden, with a cost of approximately US $300 billion per year in health care and lost productivity [6]. Thus, smoking cessation has become urgent to protect public health.

It is well-known that nicotine is addictive, which leads to smoking addiction [7]. Due to severe craving symptoms from nicotine abstinence, most smokers fail to quit smoking successfully. The success rate of smoking cessation is low (only 7.5% in 2018), even though about 55% of adult smokers are willing to quit [8]. Recognizing the addictive nature of nicotine, a growing body of studies started to examine whether very low nicotine content (VLNC) cigarettes have the potential to help smokers gradually quit smoking and its corresponding health effects. After switching to VLNC cigarettes, adult smokers reduced their smoking rate, leading to low toxicant exposure [9-13]. Other studies showed similar effects on vulnerable populations, such as individuals with mental challenges or women who are socioeconomically disadvantaged [14,15]. Several studies showed that VLNC cigarettes can reduce smoking rate and withdrawal symptoms in youth [16-18]. Furthermore, VLNC products could increase the intention to quit, the number of quit attempts, and the success rate of smoking cessation [10,19,20]. Therefore, VLNC products might provide another promising smoking cessation strategy.

Considering nicotine as the primary driver of tobacco use, in 2017, the US Food and Drug Administration (FDA) started to think about implementing a nicotine-reduction policy by reducing the nicotine level to minimize the addiction to cigarettes [21]. Recognizing the potential role of VLNC cigarettes in smoking cessation supported by extensive scientific findings, on December 23, 2021, the US FDA authorized the marketing of 2 VLNC cigarettes (22nd Century Group Inc) as modified risk tobacco products (MRTP) [22]. Therefore, these VLNC cigarette products can be marketed as having a reduced level or exposure to nicotine but cannot make any other modified risk claims. On June 21, 2022, the US government published the FDA’s proposed rule, which established the maximum nicotine level in tobacco and nicotine products to reduce the addictiveness of combustible tobacco products. This policy can potentially prevent people from smoking initiation and encourage smoke cessation [23,24]. It is estimated that with the new nicotine product standard, 33 million fewer people will become regular smokers by 2100, leading to a smoking rate of only 1.4% [24]. While this proposed rule awaits final approval, more research becomes necessary to understand the health benefits of VLNC tobacco products and their public perceptions.

With a long-standing misperception that nicotine in cigarettes is the primary agent causing cancer, a significant portion of smokers believed that VLNC products could significantly reduce the risk of cancer and other health symptoms [25-28]. This misperception might lead to a lower intention to quit smoking with VLNC products [29]. Therefore, to maximize the positive impact of the FDA’s proposed rule regulating the nicotine level, it is necessary to understand how the public perceives VLNC products so that the final policy on VLNC products can be comprehensive and accurate to avoid any potential misunderstanding. As the most popular platform for the public to share their opinions nowadays, social media provides an ideal data source for understanding how the public responds to VLNC products and related policies. Social media has been widely used to study public perceptions of different tobacco or nicotine products (such as e-cigarettes and waterpipe) as well as tobacco regulatory policies (such as the FDA flavor enforcement policy and authorization of Vuse e-cigarette products) [30-37].

This study aims to understand public perceptions of VLNC products on social media by analyzing data from Twitter (rebranded as X in July 2023). Through content analysis, we classified VLNC-related Twitter posts from December 2021 to January 2023 into different attitude groups (positive, negative, and neutral) and further grouped them into 13 major topics. Our results provide a better understanding of the public perception of VLNC products, which provides policy makers and public health authorities valuable guidance to finalize and implement the policy related to VLNC in the future.

## Methods

### Data Collection and Preprocessing

From December 12, 2021, to January 1, 2023, through Twitter streaming application programming interface (API), we collected Twitter data related to tobacco and nicotine using a set of keywords, including “nicotine,” “cig*,” “smok*,” and “tobacco.” Furthermore, using keywords related to VLNC, such as “vln,” “vlnc,” “very low nicotine,” “low nicotine,” and “reduced nicotine,” we identified the tweets related to VLNC. We removed all retweets and duplicates from the dataset [38]. In addition, we removed the commercial tweets promoting tobacco products based on keywords like “sale,” “discount,” and “deal” [39]. Eventually, we identified 3270 unique noncommercial tweets related to VLNC.

### Content Analysis

We used an inductive method for content analysis. To establish a codebook for coding all tweets, we randomly selected 300 (around 10%) noncommercial tweets related to VLNC, which were manually coded in parallel by 2 human coders (X Liu and X Lou). We classified tweets as positive, negative, or neutral based on their attitude toward VLNC. At the same time, within each attitude, we further characterized tweets into different topics. Each tweet can be assigned with more than 1 topic. There were 3 topics with a positive attitude, 7 with a negative attitude, and 3 with a neutral attitude. Between 2 human coders, we achieved a κ score of 0.75 for the attitude and 0.71 for the topic, indicating a substantial interrater agreement. Any discrepancy was resolved by a discussion with a group of 4 members (ZX, X Liu, X Lou, and DL). Based on the established codebook (Multimedia Appendix 1), the rest of VLNC-related tweets (2970 tweets) were single-coded by the 2 initial human coders (X Liu and X Lou).

As shown in Multimedia Appendix 1, topics with a positive attitude include (1) reduce cigarette consumption or help smoking cessation, discussing how VLNC can lead to less tobacco consumption or help with smoking cessation; (2) announcement of VLNC-related policies, presenting the announcement and news about VLNC with a positive attitude, such as the FDA proposed rule or the authorization of VLNC products; and (3) miscellaneous, supporting VLNC for different reasons, such as good for health, less addictive due to low level of nicotine, etc. Topics with a negative attitude include (1) VLNC is not a good method for quitting smoking, claiming that VLNC is not a good strategy for smoking cessation—instead, vaping and other methods might be a better choice; (2) VLNC leads to more smoking, thinking VLNC can lead to more smoking because smokers are trying to get enough nicotine, which can also lead to more tax income; (3) similar toxicity of VLNC as a regular cigarette, discussing that VLNC contains similar toxic chemicals as regular cigarettes; (4) human rights, claiming it is a human right to choose tobacco products and the nicotine level in cigarettes should not be regulated; (5) ineffectiveness of VLNC policy due to the black market, complaining that VLNC policy can lead to the black market on regular cigarettes since there is a great need for regular cigarettes; (6) misleading of VLNC to nonsmokers, a meta-perception concerning that the policy of VLNC can lead to the smoking initiation of nonsmokers since they might misunderstand the harm of VLNC; and (7) miscellaneous, tweets with a negative attitude for different reasons, including consumers demanding more nicotine, questioning if the VLNC policy will work, etc. Topics with a neutral attitude include (1) announcement of the FDA proposed rule of VLNC, announcing the FDA proposed rule about VLNC; (2) general information about VLNC, including general information and discussion about VLNC without any clear attitude, for example, asking whether VLNC makes sense or not, how much nicotine is in VLNC products, and sharing the general information about VLNC; and (3) authorization of VLNC products, the announcement of FDA’s authorization of VLNC products (mainly the products from 22nd Century Group Inc), and their appearance on the market.

### Statistical Analysis

We used the κ statistics to measure the interrater reliability of the 2 coders on both the attitude and topics of the tweets. We analyzed the percentage of tweets with different attitudes and topics throughout the entire study period. In addition, to gauge public opinion following the announcement of the FDA’s proposed rule regulating nicotine levels, we specifically examined the VLNC-related tweets posted between June 21, 2022, and June 24, 2022. We used a 2-proportional *z* test to compare the proportions of tweets with a positive attitude versus a negative attitude. We used the chi-square test to compare the proportions of tweets with different attitudes within the spike period versus the entire studying period. We conducted the statistical analyses using statistical analysis software R (version 4.3.1; R Foundation for Statistical Computing), with a significance level of 5% for 2-sided tests.

### Ethical Considerations

This study has been reviewed and approved by the Office for Human Subject Protection Research Subjects Review Board (RSRB) at the University of Rochester (Study ID: STUDY00006570). All tweets are publicly available and have been deidentified in this study.

## Results

**Figure 1:**
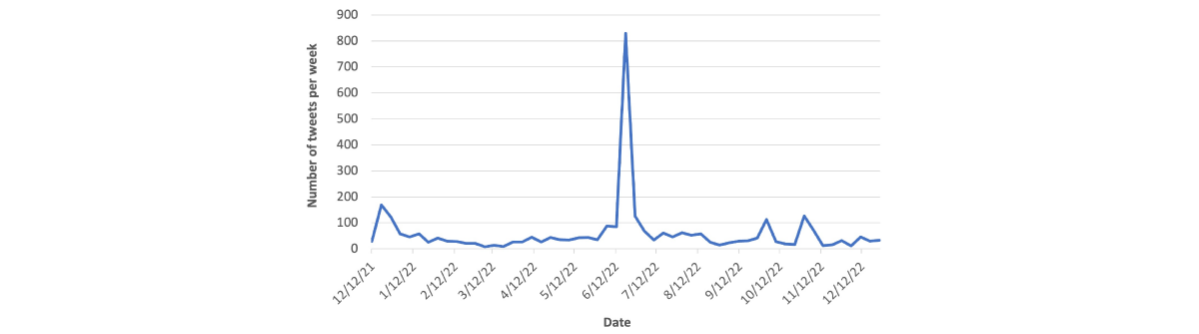
Number of very low nicotine content (VLNC)–related tweets over time.

On Twitter, we identified 3270 unique noncommercial posts related to VLNC from December 12, 2021, to January 1, 2023. As shown in Figure 1, the number of VLNC-related tweets per week was relatively low during the studying period, except there was a spike (800 tweets) between June 21 and 24, 2022, which was within 4 days after the FDA’s announcement about the proposed rule to set up the maximum nicotine level in combustible tobacco products on June 21, 2022.

**Figure 2:**
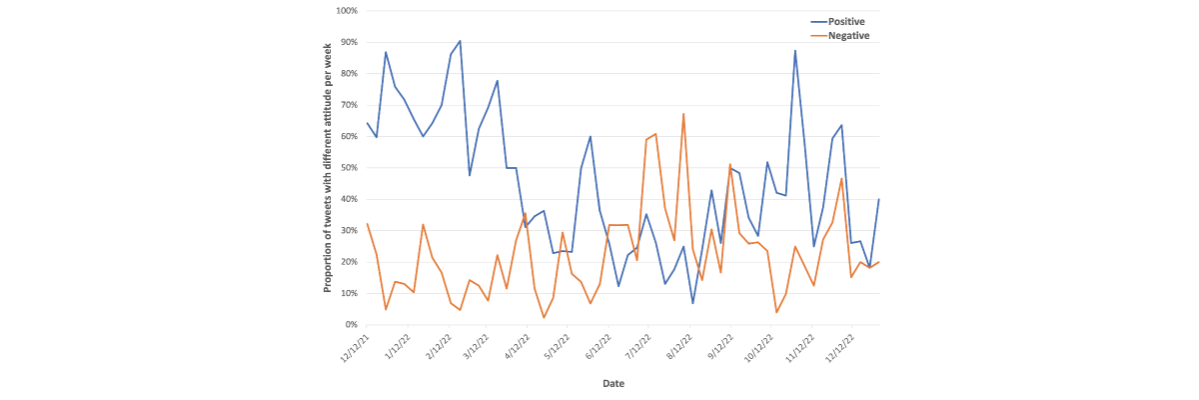
Proportion of very low nicotine content (VLNC)–related tweets with either a positive or negative attitude toward VLNC. Data with a neutral attitude are not shown.

Among the 3270 VLNC-related tweets, there were 1256 (38.41%) tweets showing a neutral attitude, 801 (24.5%) tweets with a negative attitude, and 1213 (37.09%) tweets with a positive attitude toward VLNC or related policies. Based on a 2-proportional *z* test, the proportion of tweets with a negative attitude was significantly lower than those with a positive attitude (*P*<.001). As shown in Figure 2, the proportion of tweets with a positive attitude was high at the beginning of the study period, decreased starting in April 2022, and then increased from August 2022. The proportion of tweets with a negative attitude showed an opposite pattern. In addition, the proportion of tweets with a positive attitude appeared to be higher than those with a negative attitude at the beginning and end of the study period.

As shown in Table 1, for tweets with a positive attitude, the most popular topic was “Reduce cigarette consumption or help smoking cessation” (1097/1213, 90.44%), followed by “Announcement of VLNC-related policies” (154/1213, 12.7%) and “Miscellaneous” (104/1213, 8.57%). The most popular topic for tweets with a negative attitude was “VLNC leads to more smoking” (227/801, 28.34%), followed by “Similar toxicity of VLNC as a regular cigarette” (223/801, 27.84%), “VLNC is not a good method for quitting smoking” (211/801, 26.34%), “Human rights” (86/801, 10.74%), “Ineffectiveness of VLNC policy due to black market” (24/801, 3%), “Misleading of VLNC to nonsmokers” (15/801, 1.87%), and “Miscellaneous” (66/801, 8.24%). For tweets with a neutral attitude, there were 3 topics, including “Announcement of the FDA proposed rule of VLNC” (537/1256, 42.75%), “General information about VLNC” (594/1256, 47.29%), and “Authorization of VLNC products” (125/1256, 9.95%).

**Table 1:** Major topics in very low nicotine content (VLNC)–related tweets.

Attitude and topic		Overall, n (%)	June 21-24, 2022, n (%)
**Positive (overall: n=1213; June 21-24, 2024: n=97)**			
	Reduce cigarette consumption or help smoking cessation	1097 (90.44)	62 (63.92)
	Announcement of VLNC-related policies	154 (12.7)	3 (3.09)
	Miscellaneous	104 (8.57)	32 (32.99)
	Total	1213 (100)	97 (100)
**Negative (overall: n=801; June 21-24, 2024: n=256)**			
	VLNC is not a good method for quitting smoking	211 (26.34)	37 (14.45)
	VLNC leads to more smoking	227 (28.34)	114 (44.53)
	Similar toxicity of VLNC as a regular cigarette	223 (27.84)	35 (13.67)
	Human rights	86 (10.74)	20 (7.81)
	Ineffectiveness of VLNC policy due to the black market	24 (3)	6 (2.34)
	Misleading of VLNC to nonsmokers	15 (1.87)	3 (1.17)
	Miscellaneous	66 (8.24)	19 (7.42)
	Total	801 (100)	256 (100)
**Neutral (overall: n=1256; June 21-24, 2024: n=447)**			
	General information about VLNC	594 (47.29)	98 (21.92)
	Announcement of the FDA^a^ proposed rule of VLNC	537 (42.75)	340 (76.06)
	Authorization of VLNC products	125 (9.95)	9 (2.01)
	Total	1256 (100)	447 (100)

^a^FDA: Food and Drug Administration.

Among VLNC-related tweets posted between June 21 and 24, 2022 (within 4 days after the announcement of the FDA proposed rule regulating the nicotine level on June 21, 2022), a total of 55.88% (447/800) had a neutral attitude, 32% (256/800) had a negative attitude, and 12.12% (97/800) had a positive attitude toward VLNC. The proportion of tweets having different attitudes within this spike was significantly different from those from the whole studying period (*P*<.001). As shown in Table 1, compared with the entire studying period, the proportion of topics with each attitude in this spike was different. For example, the proportion of tweets with the topic “Reduce cigarette consumption or help smoking cessation” among tweets with a positive attitude decreased from 90.44% (1097/1213) to 63.92% (62/97). In contrast, among topics with a negative attitude, the topic “VLNC leads to more smoking” increased from 28.34% (227/801) to 44.53% (114/256), whereas the topic “Similar toxicity of VLNC as a regular cigarette” decreased from 27.84% (223/801) to 13.67% (35/256) and the topic “VLNC is not a good method for quitting smoking” dropped from 26.34% (211/801) to 14.45% (37/256).

## Discussion

### Principal Findings

In this study, by analyzing Twitter posts related to VLNC, we observed a pronounced peak in discussing VLNC within 4 days after the FDA announced a proposed rule regulating the maximum nicotine level in combustible tobacco products on June 21, 2022, indicating the public awareness of potential policy related to VLNC. Furthermore, we showed that the proportion of tweets with a positive attitude toward VLNC or the proposed rule was significantly higher than those with a negative attitude. Among tweets with a negative attitude, “VLNC leads to more smoking” was the most popular topic, followed by “Similar toxicity of VLNC as a regular cigarette” and “VLNC is not a good method for quitting smoking.” In contrast, “Reduce cigarette consumption or help smoking cessation” was the most popular topic among tweets with a positive attitude toward VLNC.

Among tweets with a negative attitude, 27.84% (223/801) of them worried that VLNC cigarettes have a similar toxicity as regular cigarettes. While VLNC products have a lower nicotine level than regular tobacco products, they contain the same or nearly the same carcinogens and other toxins as in regular combustible cigarettes. Therefore, VLNC cigarettes are not safe to smoke, just like traditional combustible cigarettes. Another popular topic on Twitter is whether VLNC can help with smoking cessation. Our results showed that Twitter users were more likely to think that VLNC can help smoking or vaping cessation than that VLNC is not a good method for quitting smoking, 1097 tweets versus 211 tweets. A growing body of evidence showed that VLNC products can reduce smoking frequency, increase quit attempts, and decrease nicotine dependence and the motivation to smoke [40-43]. Therefore, VLNC products have great potential to help people quit smoking or vaping. Considering there are still some people who think that VLNC does not help with smoking or vaping cessation, effective dissemination of those scientific findings about VLNC is in great need, which can encourage the public to comply with the future VLNC policy.

One of the health concerns with VLNC products identified in our study is that VLNC might lead to more smoking, reflecting the misperception of VLNC [44]. However, previous studies did not support the notion that VLNC can lead to more smoking [45,46]. While there were 227 tweets mentioning that VLNC can lead to more smoking, there were a greater number of tweets (1097 tweets) claiming that VLNC can reduce cigarette consumption, suggesting that there is a mixed perception of VLNC by the public. Therefore, to avoid any public misperception about VLNC products, it is vital to convey clear messages about VLNC supported with trustworthy scientific evidence when the final policy on VLNC is announced in the future. In addition, we observed that 86 tweets were complaining about the regulation of the nicotine level in cigarettes since cigarette smoking is a human right or personal choice, which has been observed in other studies examining the public perception of tobacco and nicotine regulatory policies, such as the FDA’s proposed rules prohibiting menthol cigarettes or regulating e-cigarettes as medical products in Australia or the United Kingdom [33,47]. Therefore, to ensure the public’s compliance with the future VLNC policy or other tobacco and nicotine-related regulations, it is necessary to communicate effectively with the public about the health risks of tobacco and nicotine products. In this study, we observed the topic “Misleading of VLNC to nonsmokers,” which is meta-perception. This topic expressed concern about the possibility that VLNC products pose reduced health risks, which could potentially lead some nonsmokers to start using VLNC products. A possible reason for this meta-perception is the potential misunderstanding of the MRTP approval for VLNC products. Therefore, providing a detailed explanation of MRTP (especially VLNC is not risk-free) may be necessary to prevent this type of misunderstanding.

In this study, we noticed that the discussion about VLNC became very active when the FDA announced the proposed rule on setting up the maximum nicotine level, reflecting the social impacts of tobacco regulatory policy. During this period, more than half of the tweets were neutral and primarily focused on the announcement of the FDA-proposed rule on VLNC. The proportion of tweets with a negative attitude was higher than those with a positive attitude (Figure 2), largely due to the more negative sentiment toward the proposed rule on VLNC. In addition, within 4 days of the announcement of the FDA’s proposed rule, there was an increase in the proportion of the topic “VLNC leads to more smoking” from 28.34% (227/801) to 44.53% (114/256). At the same time, the topic of “Similar toxicity of VLNC as a regular cigarette” decreased from 27.84% (223/801) to 13.67% (35/256), and the topic of “VLNC is not a good method for quitting smoking” dropped from 26.34% (211/801) to 14.45% (37/256). These results suggest that the misperception about VLNC (such as VLNC leads to more smoking) increased with the announcement of the FDA’s proposed rule, which might partially stem from the inadequate and unclear communication with the public about the scientific evidence and reasoning behind the policy decision. Therefore, when tobacco regulatory policy is about to be announced, careful attention needs to be taken; for example, messages need to be clear and informative, and sufficient scientific evidence (such as VLNC products can help with smoking cessation but do not lead to more smoking) should be provided to avoid any potential misperception.

This study has several limitations. First, since Twitter users do not represent the whole population and a small group of Twitter users might be more active on social media, our findings based on Twitter data might not be fully representative. Second, basic demographic information of Twitter users is not available. Therefore, we cannot determine how different demographics (eg, gender and smokers or nonsmokers) perceive VLNC. Third, with the dynamic change of the tobacco market and regulatory policies, the public perception of VLNC is quickly evolving, which should be closely monitored in future studies.

### Conclusion

Our study revealed public perceptions of VLNC products by analyzing social media data. While many social media users think that VLNC can reduce cigarette consumption or help smoking cessation, there are several misperceptions about VLNC products, such as VLNC leading to more smoking, and VLNC is not a good method for quitting smoking. Therefore, policy makers or public health professionals must deliver effective and accurate messages about VLNC products through different media channels (including social media), which can potentially counter misperceptions and ensure the policy’s effectiveness related to VLNC products.

## Appendix

Multimedia Appendix 1 Codebook for hand-coding very low nicotine content–related tweets.

## Abbreviations

API: application programming interface
COPD: chronic obstructive pulmonary disease
FDA: Food and Drug Administration
MRTP: modified risk tobacco products
VLNC: very low nicotine content

## Data Availability

The datasets generated during and/or analyzed during this study are available from the corresponding author on reasonable request.
