# Supplementary material for: Public Perceptions of Very Low Nicotine Content on Twitter: Observational Study
Source: JMIR Form Res. 2024 Dec 4;8:e63035. doi: 10.2196/63035 (PMC11656502; doi:10.2196/63035)
Supplement: Multimedia Appendix 1 [file formative_v8i1e63035_app1.docx]

**Multimedia Appendix 1. Codebook for hand-coding VLNC-related tweets.**

| **Attitude** | **Topic** | **Description** | **Example tweets** |
| --- | --- | --- | --- |
| **Positive** | Reduce cigarette consumption or help smoking cessation | Reducing nicotine level can reduce the cigarette consumption and help smokers or vapers quit | 1. VLN studies prove smokers consume less cigarettes with VLN because it's physically impossible to get enough nicotine.  2. There is a massive amount of testing data on VLN. It all shows that it leads to fewer cigs being smoked. This product is designed to help smokers quit. It is an off ramp from nicotine addiction. |
|  | Announcement of VLNC-related policies | Announcing the policies related to VLNC with a positive attitude. | 1. Tobacco Issues Awaiting Robert Califf at the FDA.“In December, the FDA authorized the marketing of two cigarette products as “modified risk tobacco products,” or low nicotine products, to help users smoke less.”  2. Need help to #quitsmoking ? #vln #lownicotine #smokingaid #22ndcenturygroup #cigarrets #smokefree #stopsmoking 22nd Century Launching the First Reduced Nicotine Cigarette Authorized by the FDA That Helps You Smoke Less. |
|  | Miscellaneous | Support VLNC with miscellaneous topics, such as good for health, less addictive of VLNC, and no specific reason. Due to small number of tweets in each topic, they were grouped into one topic. | 1. Yeah i like lower nicotine. i want something that tastes good and takes a while to smoke but where i can smoke the whole thing and not have it feel unpleasant.  2. Now, how about we take a meaningful step towards the reduction of deaths through smoking. Support a nicotine reduction mandate. All cigs under an addictive level by 2023. |
| **Negative** | VLNC is not a good method for quitting smoking | VLNC is not a good strategy for smoking cessation. Vaping or other alternative methods are/might be better than VLNC for quitting smoking. | 1. The thing you have to ask is, what exactly are the VLNC adding to the equation? Ecigs work quite well alone, and I’m not convinced the VLNC add enough in improving cessation to be worth inhaling the toxins inherent with a combustible tobacco product?  2. That said, vaping is not only an alternative to smoking.  It can be used as a weaning tool by lowering progressively nicotine.  If VLNc works (The FDA think it will), then the same strategy with vaping will works without the harm of VLNc (it works, I see it every days). |
|  | VLNC leads to more smoking | Smokers would smoke more to get enough nicotine due to VLNC, which can increase the tax income for the government. | 1. The lower nicotine levels do not satisfy smokers, the cigarettes burn much faster; people smoke more of them to get the desired effect.  2. Cigarettes bring in more tax money. Lower nicotine in cigarettes...more cigarettes will be purchased...more tax money for government. |
|  | Similar toxicity of VLNC as a regular cigarette | Tars and other chemicals in cigarettes are making cigarettes harmful, not nicotine. VLNC can lead to similar toxic resulting from more smoking. | 1. Vapor products that millions have quit with rejected but approval of a lower nicotine cigarette that is sure to make people think it's better, yet still has all the cancer risks from combustibles. Makes sense! As always, @FDATobacco sends the wrong message &amp; fails public health.  2. New lower nicotine cigarettes with all the same high tar is NOT a safe product!!! And why would you @FDATobacco approve any cigarettes period?!? |
|  | Human rights | There should not be a regulation of nicotine level. It's personal choice and human rights. | 1. Biden Administration to Announce Plan to Lower Nicotine Levels in Cigarettes: Report #SmartNews or he could just let people smoke them as they are and stop trying to control what people do to their own bodies.  2. What if you just want democrats to stop over-regulating industry, cut excessively high taxes &amp; stop useless mandates like, low nicotine cigarettes, no soda over 32 oz's, nutrition mandates for schools &amp; fast food, etc, etc, etc. We don't need a nanny state. |
|  | Ineffectiveness of VLNC policy due to the black market | The policy on VLNC will not work due to the black market. | 1. Why would they initiate with VLNC when they will be able to access black market nicotine cigarettes?  2. Here's the reality of reducing nicotine to non-addictive levels in cigarettes. A black market for cigarettes will be created that will make prohibition look like a walk in the park. |
|  | Misleading of VLNC to nonsmokers | Posts worried that non-smoker, especially youth will try VLNC because they are advertised to be less harmful. | 1. And experimentation with VLNC might prime them to try full nicotine cigarettes in which case they could become habitual smokers.  2. LOW NICOTINE CIGARETTES COULD TURN OUT TO BE A SOURCE FOR STARTING A CIGARETTE ADDI(C)TION. YES, I AM YELLING! |
|  | Miscellaneous | Negative attitude towards VLNC with miscellaneous topics, such as consumers want more nicotine, environmental impact due to more cigarette butts, and questioning if the policy will work. But the number of tweets is small in each topic. | 1. I know. But the difference between almost no nicotine and no nicotine is not as big as some people would have us believe. Ultra lights contain 0.1 mg nicotine. I've read that VLN contain 0.05 mg nicotine. 0.1 mg feels like inhaling air. I would imagine that 0.05 mg feels the same  2. Thats the point. Noone will end smoking with low nicotine cigarettes. Its just not gonna happen. |
| **Neutral** | General information about VLNC | General information (such as the definition) about VLNC and asking questions about VLNC. | 1. VLN is genetically modifed to produce low nicotine CONTENT.  2. What do you think about very low #nicotine #cigarettes (#VLNC)? |
|  | Announcement of the FDA proposed rule of VLNC | Announcement or news about the proposed rule of VLNC. | 1.Biden Administration to Pursue Rule Requiring Lower Nicotine Levels in U.S. Cigarettes - The Wall Street Journal.  2. "F.D.A. Set to Propose Lower Nicotine Levels in Cigarettes" by Christina Jewett and Andrew Jacobs via NYT |
|  | Authorization of VLNC products | FDA authorize marketing of VLNC products from 22^nd^ Century Group | 1. FDA Permits Marketing of 22nd Century Low Nicotine Brands  2. FDA Authorizes Marketing of 22nd Century Group VLN as a Modified Risk Tobacco Product |
